# Computer adaptive testing strategies for the Edinburgh Postnatal Depression Scale (EPDS)

**DOI:** 10.1007/s00737-025-01562-5

**Published:** 2025-02-14

**Authors:** Emily F. Wong, Eynav E. Accortt, Seung W. Choi, Tiffani J. Bright

**Affiliations:** 1 Department of Computational Biomedicine, Cedars-Sinai Medical Center, Los Angeles, CA USA; 2 Department of Obstetrics and Gynecology, Cedars-Sinai Medical Center, Los Angeles, CA USA; 3 Department of Educational Psychology, The University of Texas at Austin, Austin, TX USA

**Keywords:** Perinatal mood and anxiety disorders, Postpartum depression, Computer adaptive testing, Edinburgh postnatal depression scale, Algorithmic fairness

## Abstract

**Purpose:**

Perinatal mood and anxiety disorders (PMADs) include depressive and anxiety disorders during pregnancy or postpartum and can have significant consequences for the parent, child, and family. When severe, these conditions can lead to suicide. Despite numerous policy efforts to improve screening, education, and referral structures, disparities in PMAD diagnosis and treatment still exists, particularly among racial and ethnic minorities. Computer Adaptive Testing (CAT) has been shown to improve the efficiency of screening by significantly reducing test length. This study evaluates whether applying CAT to the Edinburgh Postnatal Depression Scale (EPDS) maintains diagnostic accuracy while ensuring these methods do not exacerbate racial disparities in PMAD screening outcomes.

**Methods:**

Using real data simulation, we assessed three CAT-based short-form versions of the EPDS, derived from one-, two-, and three-factor item response theory models. We evaluated their diagnostic precision and examined potential racial disparities in false negative rates compared to the full-length EPDS.

**Results:**

We demonstrate that estimated scores from three short versions of the EPDS administered through CAT—assuming one, two, and three-factor item response theory models—are more highly correlated with the full-length EPDS measure traditionally used to make clinical decisions (*r’s* between 0.96 and 0.97) than the major depressive disorder subtest (CAT-MDD) from CAT-Mental Health (CAT-MH^®^) (*r* =.82), as previously reported. Importantly, the false negative rates of the CAT-implied diagnoses did not significantly vary between racial groups, indicating no evidence of racial bias in diagnostic accuracy.

**Conclusion:**

The CAT-based versions of the EPDS offers a promising solution for improving the efficiency of PMAD screening without sacrificing diagnostic precision or exacerbating racial groups. By reducing evaluation time, these tools could facilitate more widespread and equitable screening, enabling earlier diagnosis and treatment of PMADs across diverse populations.

## Highlights

• Short versions of the Edinburgh Postnatal Depression Scale (EPDS) tool, if administered through computer adaptive testing (CAT), provide very close approximations of the full scale but may miss a small portion of patients who otherwise would have screened positive.

• The CAT versions of the EPDS provide a closer approximation of the standard screening tool for perinatal mood and anxiety disorders (PMAD) than the CAT-Mental Health (CAT-MH^®^).

• We recommend screening for suicidal ideation after the CAT-administered EPDS for all patients to minimize the number of missed diagnoses.

## Introduction

### Overview of perinatal mood and anxiety disorders (PMADs)

Perinatal Mood and Anxiety Disorders (PMADs) encompass a range of mental health disorders, including major depressive disorder, generalized anxiety disorder, obsessive compulsive disorder, panic disorder and post-traumatic stress disorder that occur during pregnancy or up to one year postpartum (Meltzer-Brody and Rubinow 2021). A commonly studied PMAD is postpartum depression (C. Dennis and Chung-Lee 2006); Falah-Hassani et al. 2017), which affects approximately 13% of birthing parents in high-income countries (Bauman et al. 2020; Gavin et al. 2005), 20% in low-income and middle-income countries (Fisher et al. 2012), and over 25% during the COVID-19 pandemic (Sahebi et al. 2024). The estimated prevalence of postpartum anxiety disorders is approximately 10% (C.-L. Dennis et al. 2017). Despite recent national and state efforts (“Committee Opinion No. 630,” 2015; Earls 2010; Siu et al. 2016), fewer than one in four Los Angeles women are screened or educated about PMADs during prenatal, postpartum or well-child medical visits (Los Angeles County Department of Public Health 2016). Traditional PMAD risk factors include history of depression, racial discrimination, and adverse perinatal outcomes such as preeclampsia (Meltzer-Brody and Rubinow 2021). However, these risk factors often lack the specificity or sensitivity required to inform effective clinical decision-making or prevention interventions (Cox et al. 2016; Declercq et al. 2022; Sidebottom et al. 2021). Therefore, there is an urgent need for improved diagnostic and prognostic models to enable early identification and treatment of PMADs.

The consequences of untreated PMAD are not only significant for the birthing person but also for their children, partners, and familial relationships. When severe, untreated postpartum depression can lead to suicide, one of the leading causes of perinatal death (Grigoriadis et al. 2017). Beyond life-threatening consequences, even moderate PMADs can significantly impair quality of life, social functioning, and may increase engagement with more risky behaviors (Chithiramohan and Eslick 2023; Feldman et al. 2009; Miller et al. 2022; Misri and Kendrick 2008; Paulson et al. 2006; Slomian et al. 2019; Trost et al. 2017; Zelkowitz and Milet 1996). Less severe cases of PMAD can also negatively impact infant development, including motor, cognitive, language, and emotional development domains (Davis et al. 2004; Feldman et al. 2009; Fihrer et al. 2009; Grace et al. 2003; Miller et al. 2022; Misri and Kendrick 2008; Paulson et al. 2006; Tronick and Reck 2009; Zelkowitz and Milet 1996). Early detection and prevention are key to mitigating these outcomes and international guidelines emphasize the need for better identification strategies (Meltzer-Brody et al. 2018; Milgrom and Gemmill 2015; National Collaborating Centre for Mental Health and National Institute for Health and Clinical Excellence and others 2008). In recognition of this, California passed legislation in 2018 mandating that birthing hospitals properly screen, educate, and refer individuals at risk of PMAD to appropriate mental health care (Accortt et al. 2022).

### Racial and ethnic disparities in PMAD diagnosis and care

While PMADs affect individuals across all demographic groups, racial and ethnic disparities significantly worsen outcomes in marginalized communities. Women of color are at higher risk for PMADs due to systemic racism, inequities in social determinants of health, and early life disadvantages, all of which are strongly associated with mood disorders (Giurgescu et al. 2016; Mehra et al. 2020; Somerville et al. 2021). Despite this elevated risk, women from minority backgrounds are less likely to be screened for PMADs due to barriers, including limited access to care, cultural stigma, shame, and biases within the healthcare system (Abrams et al. 2009; Dennis and Chung-Lee 2006). Studies also show that low-income Black women in urban areas tend to underreport depressive symptoms on psychometric questionnaires(Chaudron et al. 2010; Tandon et al. 2012; Yang et al. 2017) despite having the same diagnosis, leading to higher rates of false negatives compared with other groups. Fear of involvement with child protective services (CPS) is one factor contributing to this underreporting, as Black children are disproportionally referred to CPS (Hsieh et al. 2021; Putnam-Hornstein et al. 2013). This highlights a critical gap in the current screening processes, underscoring the need for more culturally sensitive and accurate diagnostic tools to detect PMADs in diverse populations.

### Psychometric screening and computer adaptive testing (CAT)

One of the most widely used screening tools for PMADs is the Edinburgh Postnatal Depression Scale (EPDS) (Cox et al. 1987; Levis et al. 2020) a 10-item questionnaire designed to assess postnatal depression and anxiety (Park and Kim 2022), but it may be limited by response biases and lengthy administration. The original evaluation of the scale suggested satisfactory validity and reliability, Cronbach’s $\:\alpha\:=0.87$ (Cox et al. 1987). While effective, traditional psychometric screening can be prone to response biases associated with fixed-order tests.

Computer adaptive testing (CAT) is a method based on item response theory (IRT) models that dynamically adjusts the order of test items in real-time based on a respondent’s previous answers(Meijer and Nering 1999) and is terminated once the estimated trait (e.g., intelligence, the severity of depression) level for the respondent falls below a pre-defined threshold or after a certain number of items have been administered. This approach can circumvent response biases associated with fixed-order tests. Additionally, CAT has the potential to streamline the diagnostic process while maintaining precision. Recent advancements in CAT offer promising avenues for clinical screening. However, the integration of CAT into clinical practice must ensure it does not perpetuate racial disparities. For example, CAT-MDD from CAT-MH^®^ intelligently selects items from a larger item bank, reducing response burden while maintaining strong correlations with longer measures (*r* =.82) (Kim et al. 2016). While CAT is typically administered electronically, it is still possible to administer via paper-based adaptive tools (Chang 2015). This paper explores three short versions of the EPDS, comparing their effectiveness when administered via CAT to standard full-length measures.

### Evaluating algorithmic bias in CAT

While CAT offers the potential to improve diagnostic efficiency, it is imperative to assess the accuracy of the PMAD diagnoses made through CAT to ensure that the model does not perpetuate disparities in care. In this paper, we assess algorithmic bias in CAT systems using False Negative Rate (FNR) parity, a fairness metric designed to evaluate whether a model’s likelihood of missed diagnosis is independent of racial or ethnic group membership (Abrams et al. 2009; Chaudron et al. 2010; Dennis and Chung-Lee 2006; Tandon et al. 2012; Yang et al. 2017). Given that minority women are more likely to have missed PMAD diagnoses, achieving FNR parity is crucial to ensuring equitable healthcare outcomes.

## Methods

### Data collection

The data were collected as part of the Postpartum Depression Screening, Education and Referral Quality Improvement (QI) Initiative at Cedars-Sinai Medical Center (CSMC), located in Los Angeles, California, from February 2022 through December 2023. The dataset includes screening data from birthing individuals 18 years or older admitted to either the Postpartum Unit (PPU) or the Maternal-Fetal Care Unit (MFCU) following delivery. Exclusion criteria included individuals screened during the prenatal/predelivery period, those who experienced miscarriage or stillbirth, or individuals under the age of 18. The hospital’s Institutional Review Board approved the use of deidentified patient data for this study (Accortt et al. 2022; Accortt and Wong 2017).

In February 2022, Cedars-Sinai implemented routine screening for postpartum depression using the 10-item EPDS, which has scores ranging from 0 to 30, with higher scores indicating a greater severity of depression and anxiety. To minimize the risk of missed diagnoses, CSMC uses a more sensitive clinical cutoff of 8 or greater to indicate moderate risk for PMADs, while scores above 13 or reports of suicidal ideation indicate high risk for PMADs (Accortt and Wong 2017). All patients who screened moderate to high risk triggered referrals to social work services. All items were administered to patients via iPads, either on the day of delivery or within nine days postpartum. For individuals with multiple deliveries (e.g., twins), only the first delivery was included in the analysis. The final dataset consisted of 8,750 patient-level responses, each including EPDS scores, race, and age information.

### Statistical analysis

All analyses were conducted in R (version 4.4.0). IRT models were estimated using the mirt package and real-data simulations of CAT performance were performed using the mirtCAT package. False negative rate parity was computed using the fairness package; this metric evaluates whether the model’s likelihood of missed diagnosis is independent of racial or ethnic group membership, a critical consideration in ensuring equitable healthcare outcomes. All corresponding data and code are publicly available at https://github.com/TheBrightLab/CAT-Strategies-for-the-EPDS.

### Item response theory models

We evaluated three graded-response IRT models for the EPDS assuming three widely studied factor structures that make use of all ten items:

- One-Factor Item Model. This model assumes a single underlying factor of depression and anxiety (Cox et al. 1987). The one-factor model fit the data reasonably well: M2(15) = 791.42, *p* <.001, RMSEA = 0.08, TLI = 0.95, CFI = 0.97, with AIC = 90020.22 and BIC = 90303.29.
- Two-Factor Item Model. This model separates items into two groups: anxiety (items 3–5) and anhedonia-depression (items 1, 2, and 6–10) (Coates et al. 2017). This model provided better fit than the one-factor model: M2(14) = 486.49, *p* <.001, RMSEA = 0.06, TLI = 0.97, CFI = 0.98, with AIC = 88938.0 and BIC = 89228.15.
- Three-Factor Model. The three-factor model groups items into three categories: anhedonia (items 1 and 2), anxiety (items 3–5), and depression (items 6–10) (Coates et al. 2017). This model provided the best fit: M2(12) = 135.58, *p* <.001, RMSEA = 0.03, TLI = 0.99, CFI = 1.00, with AIC = 88101.02 and BIC = 88405.32.

### Computer adaptive testing

To evaluate the performance of CAT, we conducted real-data simulations assuming each of the three IRT models described above. The simulations estimated how patients would have been screened had CAT been used. Here, we describe the test designs for the best-performing CATs corresponding to each of the three IRT models. For the one-factor model, the starting item was selected based on maximum information (MI) and subsequent items were selected based on the maximum posterior weighted information criteria. For multi-factor models, item selection was based on the maximum (potentially weighted) trace of the information matrix (Trule). Expected a priori (EAP) estimation was used to calculate the trait scores, and the test terminated once the changes in estimated trait scores fell below 0.05. A formula-based correction for shrinkage (De La Torre and Deng 2008) was applied to all CAT-estimated trait levels to ensure accuracy.

## Results

### Patient characteristics

Patients included in the analysis were between 18 and 54 years old at the time of their delivery at CSMC (*M* = 34.26, *SD* = 4.89). The majority of patients identified as non-Hispanic white (54%) followed by Asian or Native Hawaiian or Other Pacific Islander (AAPI) (12%), Other (12%), Hispanic White (10%), Black (7%), Multiracial (4%), and Unknown (i.e., patient declined) (1%) patients. Understanding the demographic makeup of the sample is essential for interpreting the generalizability of these findings, especially given the importance of racial and ethnic disparities in PMAD screening.

### EPDS reliability and prevalence of positive screens

**Fig. 1:**
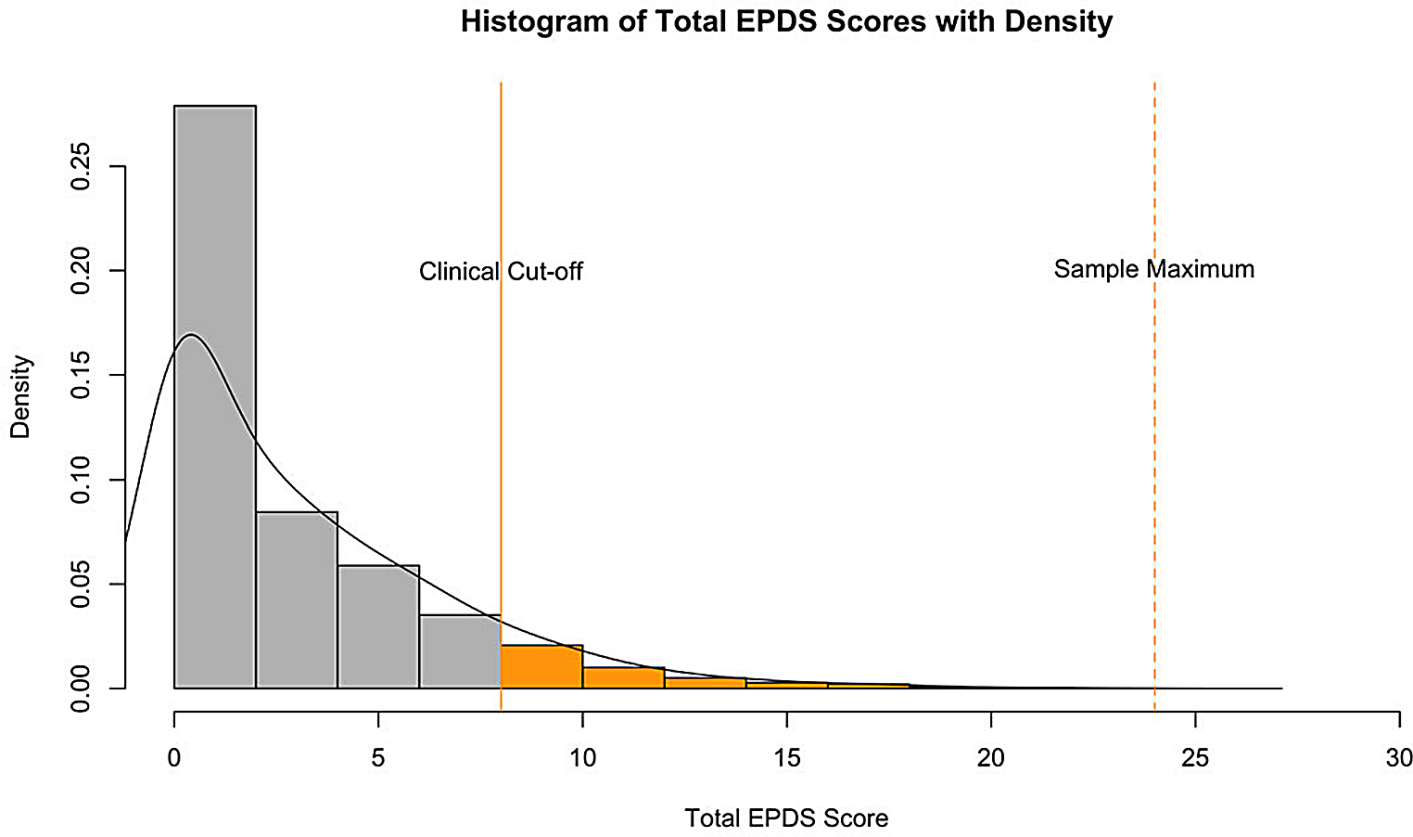
Histogram of the Total EPDS Scores with density. The solid line indicates the clinical cut-off value of 8 and the dotted line indicates the maximum score in our sample

The reliability of the 10-item EPDS in our sample was very good, with a Cronbach’s $\:\alpha\:=\:$0.81, 95% CI: [0.81, 0.82], indicating high internal consistency among the items. Approximately 11.7% of patients screened positive based on the full administration of the EPDS. Figure 1 displays the distribution of total EPDS scores in our sample, with a solid line marking the clinical cut-off score of 8.

### Number of items administered through CAT

**Fig. 2:**
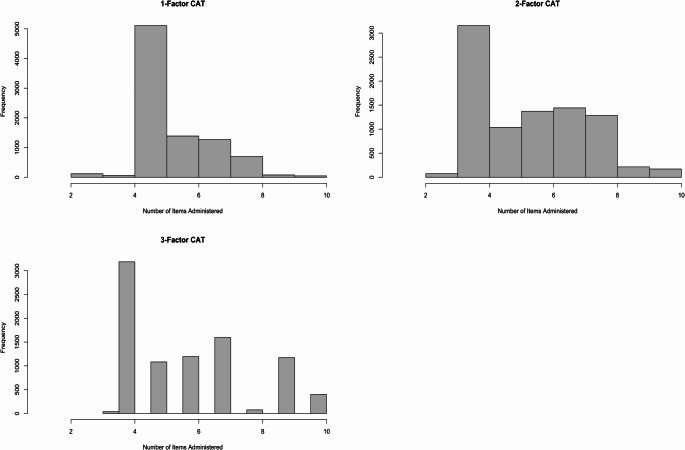
Histograms of the number of items administered for the One-Factor (upper left), Two-Factor (upper right), and Three-Factor CATs (lower left)

The use of CAT significantly reduced the number of items administered compared to the full 10-item EPDS. The median number of items administered using the one-factor CAT was 5, ranging from as few as 2 items to as many as 10. For the two-factor and three-factor CAT models, the median number of items administered was 6, with a range of 2 to 10 and 3 to 10 items, respectively. Figure 2 shows the distribution of items administered for each of the three CAT models.

### Correlation between CATs and full-test scores

The estimated trait levels from the CAT versions of the EPDS were highly correlated with those from the full bank graded response models, indicating that CAT provides results consistent with the traditional full-length version. For the one-factor CAT, the correlation between the estimated trait levels and the full EPDS was *r* =.98, *p* <.001. For the two-factor CAT, the estimated anxiety and anhedonia-depression trait levels were correlated with the full test at *r* =.86 and *r* =.97, respectively, *p*’s < 0.001. Lastly, for the three-factor CAT, the estimated anhedonia, anxiety, and depression trait levels were correlated with the full EPDS at *r* =.46, *r* =.96, and *r* =.88, respectively, *p*’s < 0.001.

**Fig. 3:**
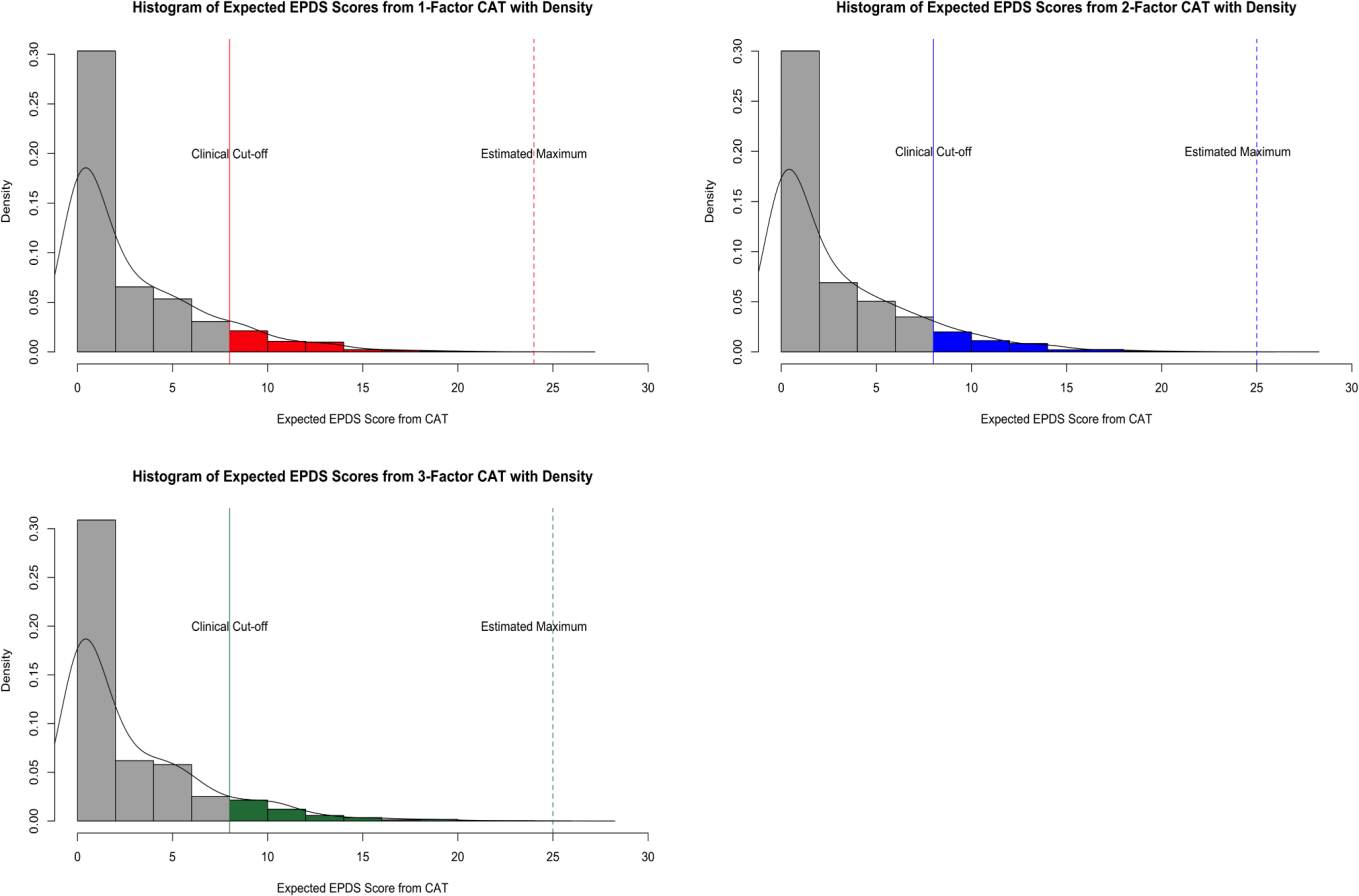
Distribution of the predicted total EPDS scores from the One-Factor (upper left), Two-Factor (upper right), and Three-Factor (lower left) CATs. The solid line on the left denotes the clinical cut-off of 8 and the dotted lines on the right denote the maximum predicted scores

The predicted total scores from each CAT model were also highly correlated with the observed EPDS scores. The correlation was *r* =.963, 95% CI [0.962, 0.965], *p* <.001 for the one-factor CAT, *r* =.969, 95% CI [0.968, 0.970], *p* <.001 for the two-factor CAT, and *r* =.965, 95% CI [0.963, 0.966], *p* <.001 for the three-factor CAT. These high correlations suggest that CAT provides highly reliable estimates of a patient’s PMAD risk compared to the full EPDS. Figure 3 shows the distribution of the predicted total EPDS scores from each of the three CATs, with a solid line marking the clinical cut-off score of 8.

### CAT-implied diagnosis

In clinical practice at CSMC, patients are considered high risk for PMAD if they score 13 or higher on the EPDS or endorse the suicidal ideation item. A score between 8 and 12 indicates moderate risk, while scores below 8 are classified as low risk. Patients classified as moderate or high risk are referred to social work services for further intervention. However, CAT algorithms may skip harder items (such as the suicidal ideation item) for patients who provide low scores on easier items. To address this, we evaluated each patient’s expected total score from the CAT alongside their response to the suicidal ideation item to determine their overall classification. If the expected total score was greater than or equal to 8, or if the suicidal ideation item was endorsed, the CAT-implied classification was considered “positive.”

### Predictive value of CAT-implied classifications

Approximately 13% of patients would have screened positive for PMAD based on the one and two-factor CAT-implied classifications, while 12% would have screened positive based on the three-factor CAT-implied classification. The negative predictive value (NPV), which indicates how likely a “negative” prediction is to be truly negative, was approximately 98.8% for the one-factor and three-factor CAT models and 99.3% for the two-factor CAT model. This high NPV suggests that CAT is highly accurate in identifying patients who do not require follow-up care. The positive predictive value (PPV), which indicates how likely a “positive” prediction is to be truly positive, was 84.2% for the one-factor model, 86.7% for the two-factor model, and 88.6% for the three-factor model. These results demonstrate that CAT-based models maintain strong clinical utility by accurately identifying patients who need intervention.

### Bias evaluation

We conducted chi-square tests to evaluate whether the FNR of the CAT-implied classifications varied across racial groups. For the one-factor CAT, the overall FNR was approximately 9%, 95% CI [7%, 11%], with no significant difference between racial groups, 𝜒^2^(6) = 2.51, *p* =.867. For the two-factor CAT, the overall FNR was 5.5%, 95% CI [4%, 7%], with no significant variation between racial groups, 𝜒^2^(6) = 5.95, *p* =.429. Similarly, the three-factor CAT had an overall FNR of 9%, 95% CI [7%, 11%], with no differential false negative rates between racial groups, 𝜒^2^(6) = 2.24, *p* =.897.

**Fig. 4:**
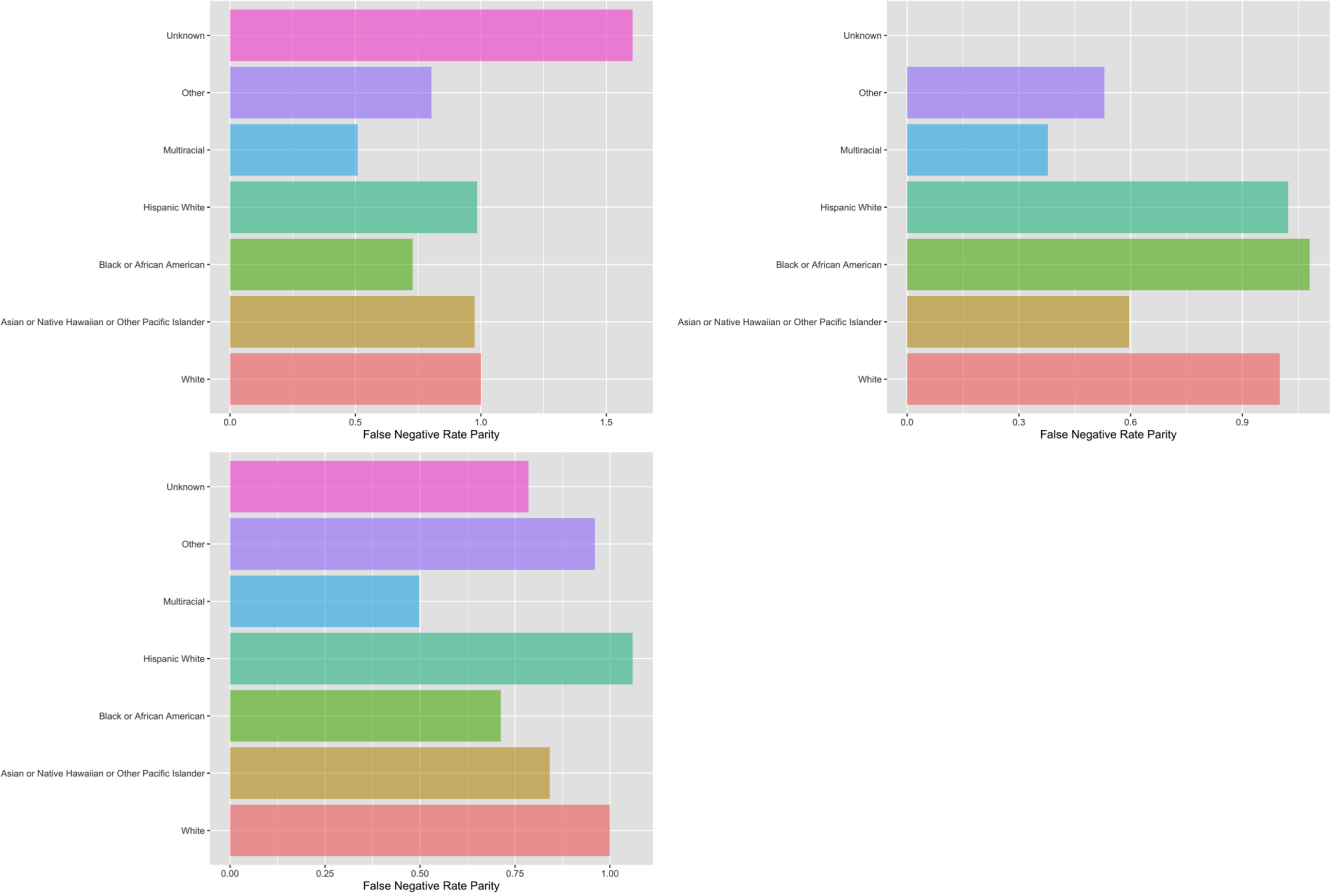
FNR parity assuming “White” as the reference group for the one-factor (upper left), two-factor (upper right), and three-factor (lower left) CATs

These findings suggest that the CAT models are not likely to introduce racial bias in PMAD screening, an important consideration in ensuring equitable healthcare outcomes across diverse populations. Figure 4 shows the FNR parity using non-Hispanic White (i.e., “White”) patients as the reference group.

## Discussion and conclusions

The current study evaluated whether three short-form versions of the EPDS administered through CAT can improve the efficiency of PMAD screening without significant loss of diagnostic precision and whether the algorithm is at risk of known reproducing health disparities. The results demonstrated that the scores from all three CAT versions were highly correlated with the full EPDS scores (*r*’s between 0.96 and 0.97), outperforming previous CAT measures like CAT-MH (*r* =.82). This high correlation suggests that CAT can reliably reduce the number of items administered, cutting evaluation time nearly in half for most patients, while maintaining diagnostic accuracy. The results also demonstrated that the CAT-implied diagnoses from all three models yield similar false negative rates across racial groups. This suggests that this implementation of CAT is unlikely to reproduce known health disparities in which PMAD diagnoses for patients of color, particularly Black and Hispanic patients, are more likely to be missed.

These findings have significant implications for public health and clinical practice. By reducing the time required for assessments, the CAT versions of the EPDS can make routine mental health screening more feasible at critical touchpoints in maternal care, including prenatal visits, postpartum checkups, and well-baby visits. Implementing CAT-based EPDS could enhance the likelihood of integrating mental health screenings into standard workflows, thereby increasing the percentage of people screened during these visits. Early detection of PMADs through these enhanced screening methods is critical for improving maternal and child health outcomes. Furthermore, addressing PMADs early can reduce some of the consequences of untreated PMADs discussed earlier in the paper. Importantly, the demonstrated efficiency and equity of the CAT-based EPDS screening tools have implications for scaling and policy development. These tools could become part of a broader equitable mental health care strategy that includes AI-based tools for identifying individuals at risk of developing PMADs (Wong et al. 2024).

In conclusion, the CAT-based EPDS offers a promising approach to improving the efficiency, equity, and accessibility of PMAD screening. By addressing barriers to routine mental health evaluation and reducing diagnostic disparities, this approach has the potential to significantly enhance early detection, prevention, and treatment of PMADs, ultimately contributing to better health outcomes.

### Limitations and future directions

While the results highlight the efficiency and accuracy of CAT-administered short-form EPDS versions, there are several limitations to note. First, between 5.5% and 9% of patients who would have screened positive using the full EPDS may be missed by the CAT versions. This suggests that while CAT reduces screening time, there is a trade-off in diagnostic precision that needs further exploration. Future studies should investigate how these missed cases impact clinical outcomes and whether improvements to CAT algorithms can mitigate this limitation. Another important limitation is the need to screen for suicidal ideation in all patients. Since CAT may skip the suicidal ideation item for patients who respond with low severity on previous items, it is critical that this item be presented to all patients, regardless of their prior responses. Ensuring this safety measure is in place would reduce the risk of missing high-risk individuals. In addition, the reliance on electronic devices for administering CAT poses accessibility challenges, particularly in resource-limited settings. While hospitals like Cedars-Sinai have the infrastructure to administer iPad-based screenings, other institutions may lack the necessary technology. Future research should explore alternative, low-cost solutions for administering CAT, such as mobile phone applications or paper-based adaptive tools (Chang 2015). Overcoming these technological barriers is essential to ensure that CAT can be adopted in a wide range of healthcare environments.

Furthermore, a significant challenge to implementing CAT is the lack of training and awareness among healthcare providers. Without proper education on how to use CAT, its benefits may not be fully realized. Developing training programs for professionals, as well as partnerships with health technology companies, could facilitate the adoption of CAT. In addition, grant funding could help reduce the financial burden on healthcare providers in resource-limited settings. Lastly, while the EPDS is a widely used tool for PMAD screening, future studies should compare its diagnostic efficacy to other screening tools, such as the 4-item CAT-MDD, to determine if shorter forms provide similar accuracy without increasing the rate of missed diagnoses. Future research should also explore the utility of CAT in different populations, such as rural communities or culturally diverse groups, to ensure its generalizability.
